# The Effects of Noise on Cognitive Performance and Helplessness in Childhood: A Review

**DOI:** 10.3390/ijerph20010288

**Published:** 2022-12-24

**Authors:** Maud Dohmen, Ella Braat-Eggen, Astrid Kemperman, Maarten Hornikx

**Affiliations:** 1 Building Acoustics Group, Department of the Built Environment, Eindhoven University of Technology, P.O. Box 513, 5600 MB Eindhoven, The Netherlands; 2 Department of the Built Environment, Avans University of Applied Sciences, 5037 DA Tilburg, The Netherlands; 3 Urban Planning and Transportation Group, Department of the Built Environment, Eindhoven University of Technology, P.O. Box 513, 5600 MB Eindhoven, The Netherlands

**Keywords:** environmental noise, children, cognition, learned helplessness, motivation

## Abstract

Environmental noise affects our daily functioning in many ways, and the cognitive, motivational, and emotional effects of noise are intertwined. Our task performance under noisy conditions depends on our ability to cope with the noise and our cognitive resources. The process of (failed) coping may wear us out cognitively, lead to learned helplessness, and, consequently, alter the motivation to persist in a task. The direct effect of irrelevant sounds on cognitive functioning in children is relatively well-established, however, the research on the framework of learned helplessness is limited when it comes to children. Learned helplessness can give more insight into effects of environmental noise on learning and child development and how the effects of short-term and long-term exposure interact. A systematic literature review is performed to assess to what extent the current evidence addresses the (interaction) effects of the sound environment on cognition and learned helplessness as measured by motivation in children and young adults up to the age of 21. The search resulted in 8 included papers that addressed both cognition and learned helplessness in their research. The included papers study children between 8–13 years old and show evidence for a relation between environmental noise, cognition, and helplessness individually, but none study a possible interaction. Based on the individual study designs, it could be hypothesized that cognitive fatigue may play a role in the interaction. Studies that conducted motivation tasks after cognitive tasks found stronger effects than those that conducted tasks in a random order. More research is needed using the same methods in different age groups to further assess the interaction between cognition and learned helplessness in relation to the sound environment.

## 1. Introduction

The physical environment impacts our health and well-being in many ways. Noise in particular may affect cognitive processing, mental health, and motivation to complete tasks or activities [1,2]. Children are more vulnerable to the negative effects of noise compared to adults because of their developing cognitive skills, lesser capability to anticipate stressors, and developing coping repertoire [3,4]. Therefore, the negative effects of noise in crucial developmental stages in a child’s life can have lasting effects.

Several frameworks exist to explain the mechanisms behind the effects of noise on cognition, mental health, and helplessness. First, addressing cognition and the mechanisms that focus on noise and the effect on cognitive performance includes (1) the order processing account or changing-state hypothesis, (2) the phonological or semantic processing account, and (3) the duplex theory of auditory distraction [5,6].

The first mechanism, the changing-state hypothesis, applies mainly to tasks involving order processing, e.g., serial recall [5]. Interference is caused when background noise is composed of a series of sounds with acoustic variation [6]. Here, the sound itself contains order information and interferes with the processing of the order processing tasks. The second mechanism is related to working memory. The working memory maintains, stores, and manipulates incoming information [5,6]. Visually presented words or numbers are rehearsed phonologically in the working memory. When background noise is present, and both visual and auditory input need to be processed at the same time, this can cause interference [5,6]. This is especially true for sounds with semantic meaning, e.g., speech in a language you understand [6]. Following this theory, people having a better-trained working memory are expected to perform better [5]. Children are still developing their skills and are therefore expected to be more affected by noise. The last theory on cognitive performance, the duplex theory of auditory distraction [3], attributes the interference of noise in a task to interference by process and attentional capture. The interference by process part has similarities with the phonological or semantic processing account. The difference is that the former states that distraction occurs because the task and the noise depend on the same processes, and the latter states that distraction depends on the semantic properties of the sound [6]. Attentional capture occurs when a noise deviates from the auditory context [3,6]. For children whose attentional skills are still developing, the effects of noise on task performance can be greater [5,7]

In addition to cognitive functioning, two other frameworks focusing more on the development of stress and coping with stressors are addressed, namely, the environmental stress model [8,9] and the learned helplessness theory [10]. The environmental stress model focuses on the cognitive resources that are needed to appraise and cope with the incoming sound and the stress that emerges when coping is unsuccessful. The accumulation of stress due to unsuccessful coping may lead to mental health problems in later life. The learned helplessness theory concerns a psychological state that is the result of continually encountered (aversive) events that one can do nothing about [10]. The aversive events that are central to this concept can be anything from social to physical stressors, but there are indications that environmental noise exposure plays a role [11]. Learned helplessness is more than stress, it is a broad psychological concept and is centered around the perception of control [10]. Learned helplessness is defined as the state that occurs when “an organism learns that its behavior and outcomes are independent, and that this learning produces the motivational, cognitive, and emotional effects of uncontrollability” [10]. So, it can manifest in three possible deficits: motivation, emotion, and cognition [12]. The motivational component, as described by Abramson et al. (1978), consists of “retarded initiation of voluntary responses” [11]. Knowing that one’s behavior will not affect the outcome, one does not initiate the behavior. In relation to task performance, this is most commonly measured as task persistence [11] and is manipulated by giving the participant an unsolvable task.

The emotional effects of learned helplessness include depression or a depressed emotional state. The pathway to this state is not per se through the expectation of uncontrollability alone. It needs to be paired with the expectation of the uncontrollable outcome to be aversive, to lower self-esteem, and cause internal attributions for failure [11].

Cognitive effects of learned helplessness include the fact that it is difficult to learn that a failed coping mechanism or failed behavior in one situation can be helpful in another, i.e., the generalization of perceived helplessness [8]. In addition, it can be speculated that impaired cognitive performance can initialize the above helplessness deficits. Failure at a cognitive task because of lowered performance due to noise could be a trigger.

The severity of these deficits depends on the individual’s locus of control and their attribution of failure. An individual’s locus of control can be external or internal. People with an external locus of control tend to believe that outcomes are not caused by their own actions but by luck, chance, or fate [11]. Those with an internal one do believe that they can influence an outcome [11]. While externals are more vulnerable to helplessness [12], both types can experience it depending on what they attribute their failure to. Internal attributions and stable factors, such as lack of skill or intelligence, are more likely to cause learned helplessness symptoms [11,13]. In relation to noise, important factors are whether or not you are able, or perceive to be able, to turn off the noise and whether you believe that you can accomplish a task under noisy conditions. If you believe you do not have the skill (internal attribution) to accomplish the task under noise, you are more likely to fail and feel helpless.

In this review the measure of motivation will be central. It is the most common measure of learned helplessness [11] and could be related to task performance, and has been proven to have a relationship with noise exposure in the past. Evans and Stecker have reviewed the effect of noise on motivation, and they found that environmental noise, both in-situ (short-term, during a task) and chronic (long-term, daily exposure at home or at school), can affect the motivational components of helplessness. They also concluded that more research is needed to determine if learned helplessness in the form of motivation can be a viable mediator in the noise exposure–mental health relationship [12].

Glass and Singer (1972) performed many experiments with noise as a stressor (in-situ) [13]. One type of experiment with a quiet or noise pre-treatment followed by a motivation test in a quiet condition showed that exposure to noise prior to the motivation test decreased task persistence afterward [12,13]. In another type of experiment, they found that when unpredictable and uncontrollable noise was presented during the task, persistence was also decreased. This could, however, be mediated if the participants were instructed that they could terminate the noise if they would like to [13]. Even though this was not true, they had perceived control, and the task persistence increased.

From the above it can be seen that the cognitive, motivational, and emotional effects of noise are related. Our task performance under noisy conditions depends on the task at hand, the noise conditions the possible interference of noise with cognitive processes, and our ability to cope with the noise [3]. Coping responses may be aimed at directly altering the stimulus (fight or flight) or relieving the emotional impact of the stimulus (denying, ignoring). The response is influenced by the availability of resources within the individual (capacity to cope) or from the environment (ability to move away, switch off the source) [9]. The individual resources, i.e., cognitive resources, need to be divided between appraisal of the source, coping, and the task at hand. When coping has failed, i.e., the source cannot be altered or emotional impact cannot be relieved, it wears us out cognitively. The cognitive resources that are left then need to be used to process the task while also processing the background noise. Motivation to keep doing this may depend on the state of helplessness and our previous experiences. When past experience has learned that failure at a cognitive task is inevitable, motivation to try and persist is lessened. So, cognitive fatigue can alter our task performance and past failure may decrease our efforts to succeed in the future. The direct effect of noise on cognitive functioning in children is relatively well-established and the negative effects of noise are evident [3,7,14,15,16,17]. However, research on the framework of learned helplessness in relation to noise is limited when it comes to children. Still, there are indications that children chronically exposed to higher noise levels experience more after-effects, such as learned helplessness, than their less exposed counterparts [12]. Additionally, to our knowledge, little research has been done into how the cognitive and learned helplessness effects of noise could be related.

It is known that chronically exposed children are more vulnerable to learned helplessness [12], possibly due to less perceived control over their environment. Chronically exposed children also perform worse on cognitive tasks [7,14,15,16,17]. Understanding how cognitive function and learned helplessness interact can give more insight into the effects of environmental noise on learning and child development. Additionally, because learned helplessness also has an emotional component, understanding this phenomenon could give more insight into the mediation of learned helplessness in the noise exposure and mental health relationship as well.

Therefore, the aim of the present paper is to review to what extent the current literature has found evidence on the interaction effects of cognition and learned helplessness (measured as motivation) in regard to the sound exposure of children and young adults up to the age of 21.

The main research questions are how does the sound environment influence the cognitive performance and vulnerability to learned helplessness in childhood to young adulthood, and how are cognitive and helplessness effects related?

This paper is structured as follows: Section 2 outlines the methodology of the literature review. Section 3 summarizes the search results, the risk of bias assessment of the included papers, and describes the results of the included papers per conducted cognitive and learned helplessness test. Section 4 discusses the results, and Section 5 presents the main conclusions of the systematic review.

## 2. Materials and Methods

### 2.1. Search Strategy and Selection

A systematic review was completed in October 2022 of the Scopus, PubMed, and psycINFO databases using the title, abstract, and keywords based on the following search query:

(child* OR adolescent* OR student*) AND (noise OR “sound environment” OR soundscape OR “acoustic environment”) AND (cognitive OR cognition OR helplessness OR motivat*).

The population under review was children and young adults up to the age of 21. This broad age range, throughout the whole of childhood, was chosen to compare the possible effects over the course of a lifetime. Studies identifying their samples as “students” were also considered, unless the age range was clearly specified to be above 21 years.

The papers had to be related to the sound environment and/or noise exposure and perform both cognitive and helplessness (motivational) tests. The research terms “cognition” and “helplessness” were separated by “OR” because the results may have been published separately or because a cognitive or helplessness test may have been performed and not mentioned in the title or abstract.

The search terms were selected by first assessing a larger group of search terms. The terms were selected if they resulted in added papers (for example “sonic environment” returned nothing additional so it was removed) and if those papers were relevant. For example, by isolating the term “sound,” the return was very large, running in the 20,000 range because “sound” is also an adjective. The search term was further specified to “sound environment” for this reason.

The papers were reviewed in three rounds based on their title, abstract, and full text. The review was performed by the first author; in cases of uncertainty, the second author was consulted. From the title, the connection to either the sound environment, cognitive performance or capacity, or helplessness must be clear. In addition, the text must have been available in English and been peer reviewed; book chapters and conference papers were excluded.

In the second round, the abstracts were assessed on their relevance. The study itself had to contain tests on the influence of sound on cognitive performance and learned helplessness. Consequently, learned helplessness studies that did not use noise in relation to performance on a task were excluded. (In learned helplessness studies helplessness is often induced by manipulating the participants perception of control using the “shuttlebox test”. This test is focused on the participant trying to terminate a noise and tests the effect of perceived control, but is not focused on the effect of the noise itself.) In addition, the subjects in the study were within the age range of 0–21 years old, had no hearing problems, and were otherwise healthy individuals.

In the third round the full text was reviewed. Each study should contain a control or reference group, and the sound environment should have been measured, modelled, or induced in a controlled manner. Finally, papers should include a clear description of the performed tests or use standardized tests. In regard to cognitive tests reading speed tests, auditory tasks and school grades were excluded. Papers were excluded as well when it was not clear if the measured effect was due to the noise or other stimuli not originating from the sound environment.

The exclusion of the above cognitive tasks was based on the fact that they might measure different elements beside cognition or did not measure cognition to a large extent. For example, reading speed tests told nothing about the comprehension of the text or retention. In addition, tests that concerned an auditory task that focused on speech-in-noise comprehension were also excluded, because they depended on the hearing and perception of the participant. The mechanisms described above concern the interaction between auditory and visual processing, and auditory tasks would focus only on the former. Furthermore, school grades were not accepted as a cognitive parameter as they might differ between schools and countries.

In regard to testing learned helplessness, standardized tests do not exist to our knowledge. However, the most common method to quantify the vulnerability to learned helplessness in a study is through measuring task persistence [11]. The primary goal of these tests is to give participants an impossible task and observe whether they persist in trying to complete the task or give up. The sequence of unsolvable and solvable puzzles in the Glass and Singer experiments [13] or adaptations thereof are the most established example of this.

### 2.2. Study Quality

The risk of bias was assessed in the selected studies using a checklist as used by van Kempen et al. [18] developed by the WHO [19]. It includes (i) information bias due to exposure assessment, (ii) bias due to confounding, (iii) bias due to selection of participants, (iv) information bias I due to health outcome assessment, and (v) information bias II due to health outcome assessment. For each study, the evaluation was carried out by the first author. Table 1 shows the scoring criteria.

## 3. Results

### 3.1. Search Results

**Figure 1:**
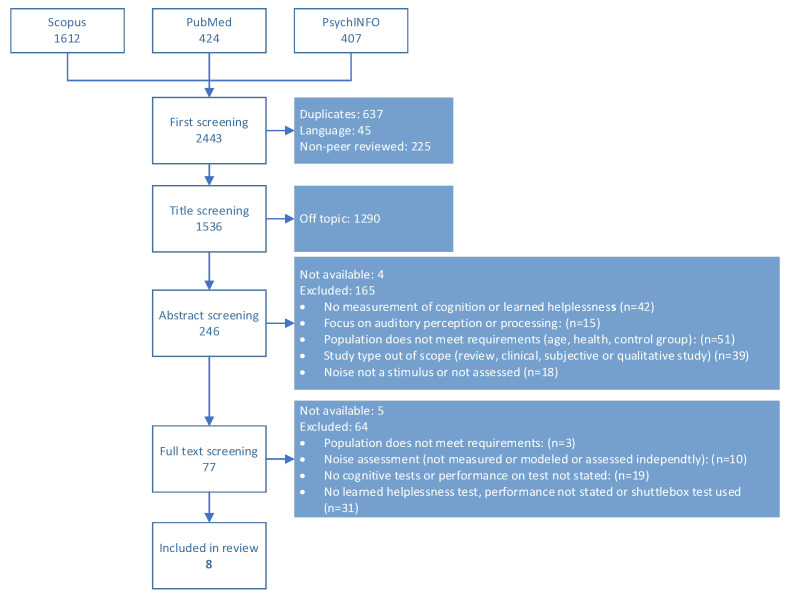
Selection process of the systematic review.

Figure 1 shows the selection process and the number of included and excluded papers per round. From 2443 initially found papers, 8 were selected to be included in the review—these papers concerned 4 different studies. Information was extracted related to the study design, sample characteristics, the noise source under consideration, noise levels, and the type of cognition and helplessness test. The eight selected papers are summarized in Table 2, grouped by individual study. For each study, the following aspects are presented: study design, sample characteristics, including gender, where available, noise source under consideration, noise levels, and the type of cognitive and learned helplessness tests. If the study design was longitudinal, the measurement waves are labeled “wave n.” The number of measurement waves and the time between them are indicated in Table 2.

### 3.2. Study Quality

Two out of the four studies were rated as having a high risk of bias, see Table 3. An important reason for risk of bias was due to participant selection. In order to score “low” on risk for bias due to participant selection, the participation rate had to be higher than 60%, and the participants should have been randomly sampled. In such cases when schools or homes were previously selected based on their exposure and matched to controls in unexposed areas, the sample was not random anymore. Although it could be argued that the quality of evidence could be increased when dose-response gradients are used, the rating was kept “high” due to the categorical use of exposure in the results (noise–quiet) [27]. The LA study [20,21] also had a high attrition rate, and in the Munich study the response rate was low, and the attrition rate study was unclear.

The matching process between the noise-exposed groups and their controls did make sure that the bias due to confounding was low. All participants were matched for age and social status, and, in the analysis, (were relevant) confounders were adjusted for.

Another factor, which two studies scored “high” on, was exposure assessment. The LA study [20,21] only performed two 1-hour measurements in the classroom and reported only the peak and L33 levels, which can be highly variant depending on the situation when measuring. The Munich study [22,23,24] conducted 24 h measurements outdoors at the location. According to the guidelines, measurements only have a low risk of bias when conducted for a week. The ALPINE [25,26] study scored a low risk of bias on noise measurements due to the use of sound exposure modeling, according to national guidelines, and the verification of those guidelines with measurements. The Heathrow study [2] also scored low bias on noise exposure assessment due to the use of contour maps that were based on measurements in the airport area from June to September and models based on aircraft movement, route, noise generation, and sound propagation data for 16 hours of the day [28].

Bias due to a non-blinded outcome assessment was judged based on any elaboration of the authors noting if there was anything done to avoid bias due to knowledge about the study. In the LA and Munich study this was not discussed, nor was it stated how the participants were informed about the study. In the ALPINE study, the experimenter was blind to the noise condition of the child. In the Heathrow study, the participants were informed that the study was about the general environment to avoid any extreme answers on the noise questions. The bias due to non-blinded outcome assessment is more relevant in the case of experiments rather than the studies under discussion here. However, regardless of the inclusion of this criteria, the overall risk of bias rating would not change.

### 3.3. Descriptive Results

The eight included papers can be grouped under four different studies: the Los Angeles (LA) noise project, the Munich airport study, the Heathrow study, and the ALPINE project. The LA project and the Munich study are of a longitudinal design; the Heathrow and ALPINE study are cross-sectional.

The participants of all the studies were primary schoolchildren in the ages 8–13. The noise source of interest was aircraft noise either at home or at school in the LA project, Munich, and Heathrow study, and in the ALPINE study, it was rail and road traffic noise at home. The main direction of the effects of noise on cognitive performance and helplessness (measured in motivation) are given in Table 4.

The type and number of cognitive tests conducted varies per study but included tests for attention, reaction time, reading, long-term and short-term memory, and intentional and incidental memory. The learned helplessness tests were aimed at the component of motivation using a series of unsolvable and solvable puzzle tasks (with puzzle pieces or line diagrams). The Munich study also questioned its participants on their attribution for their failure to solve the unsolvable puzzle. The ALPINE study included questions for attribution of failure, but these were not reported in the papers. The different tasks are discussed separately below.

#### 3.3.1. Cognitive Tasks

- *Visual search task*

As indicated in Table 4, a visual search task was performed in the LA, Munich, and ALPINE study. The type of visual search tasks used, the noise conditions during the task, and the performance of the different groups can be seen in Table 5. The LA study found that the exposed group performed better when the number of years enrolled at the school where measurements took place was less than two. It is suggested by the authors that there is an increased ability for noise-exposed children to tune-out the noise as a coping mechanism, which aids the performance of auditory distraction tasks [20]. This advantage disappears when the years enrolled in school increase.

The Munich and the ALPINE studies did not find any significant differences between groups on the visual search task [22,23,25]. Whereas the ALPINE study attributed the lack of effect to the low level of task difficulty and, therefore, the low cognitive effort needed to complete the task [25], the Munich study did not speculate on a possible reason.

Overall, the performance of the task was low in the Munich study compared to the others. The tasks were different from each other in both the type of task and the noise during the task and may differ in difficulty, so this might explain the difference in performance.

- *Reaction time task*

The type of reaction time tasks used and the noise conditions during the task can be seen in Table 6. Only the Munich study performed a reaction time task. In the first wave there was no difference between the groups or between the conditions under which the task was performed. Over time, there was a significant airport × group × wave interaction effect *p* = 0.004 [22], meaning that the differences between the old and new airport groups, the exposure groups at these locations, and the measurement time were significant. The number of errors during the task did not contribute to the interaction, only the reaction time did. The noise-exposed school group at the old airport location was slower than the control group at wave 2 (6 months after move) *p* = 0.026 and at wave 3 (18 months after move); the new airport-exposed group was slower than its control group *p* = 0.039 [22].

- *Memory tasks*

Memory was measured in multiple ways: short-term (or working memory), long-term, intentional, and incidental. The type of memory tasks used and the noise conditions during the task can be seen in Table 7.

In the short-term memory task in the Heathrow study, no significant differences were found between the exposure groups [2]. The results of the short-term memory task in the Munich study across different waves were not consistent. At the old airport location, there was a significant group × wave interaction effect *p* = 0.004; the noise-exposed group performed worse than the controls, and this performance reached the level of the controls after the airport was relocated. At the new airport location, no differences between groups across waves were found [23].

The long-term memory task in the Munich and Heathrow studies was the same in regard to the type of test used; the execution was, however, different. The Munich study used noise during the retention period, while Heathrow used no additional noise. After a day (Munich) or a week (Heathrow), long-term memory was tested using six multiple-choice questions and three recall questions. In both cases, children from the noise-exposed groups performed worse than those in the control group. The longitudinal results of the Munich study showed a significant airport × group × wave interaction *p* = 0.015 [23]. The difference between groups at the old airport location before the move was marginally significant *p* = 0.062 [23], but not any more after the move. In the new location, the difference emerged at wave 3, 18 months after the move, *p* = 0.007 [23]. Therefore, it can be hypothesized that a detrimental effect of noise on memory develops over time but can also recover when the noise source is removed.

Comparing the scores on this test, we see a smaller difference between the noise and the control group in the Heathrow study compared to the first wave in the Munich study. The exact scores for the other waves are not presented in the paper. The overall performance of both groups is also better in the Heathrow study, even though this study had a longer time between reading and testing.

The ALPINE study had a different take on memory tasks and assessed intentional versus incidental memory. For both these tasks, chronic noise exposure significantly worsened performance [25].

- *Reading tests*

The type of reading tasks used was the Biglmaier standardized German reading test [29] (Munich study [22]) and the Suffolk Reading scale level 2 (Heathrow study [2]) conducted without any additional noise during the task. The scores for the test are not discussed in this review because the measurement scales of both tests are very different. In both studies, significant differences were found between the exposed and the control groups. In the case of the Munich study, this was only true for the most difficult parts of the word part of the tests. There was a significant airport × group × wave interaction of *p* = 0.007. In the first wave, the noise-exposed group at the old airport performed significantly worse than its controls *p* = 0.009 [23], but not in waves 2 and 3. The new airport had only a marginal difference between the exposed group and its controls in wave 3 with *p* = 0.074 [23] and none in waves 1 and 2. The differences in the prose part of the German test were less pronounced, with significant differences only in the first wave between the old airport groups; weak airport × group × wave interaction effect *p* = 0.118 [23].

In the Heathrow study, the difference in performance between the groups was equal to a 6-month delay in reading ability. This effect remained when the results were adjusted for social class, deprivation, age, and mean language at home [2].

#### 3.3.2. Motivation Tasks

To measure the vulnerability to learned helplessness, two types of motivation tasks were used: line diagram puzzles and geometric puzzles. The line diagrams consisted of animal line drawings that needed to be traced without lifting the pencil or redrawing a line. The geometric puzzles consisted of the same nine pieces and required the child to fill in a template of a familiar shape: a circle (solvable), a triangle (unsolvable), and a square (solvable).

The two methods differed regarding time, measuring indicators for helplessness, and pre-treatment. The geometric puzzle task started with a pre-treatment task of a solvable or an unsolvable puzzle, which the participants were allowed to work on for 2.5 min. The idea was that an unsolvable pre-treatment would decrease motivation and induce a form of helplessness. This unsolvable test was followed by a solvable test puzzle, which was the same for all participants. They worked on this puzzle for 4 min, and the measured indicators were whether the test puzzle had been solved and whether the child was persisting or giving up before the 4 min were over. The focus was on the second solvable task with either an insolvable or solvable pre-treatment.

In the second wave of the LA study, the children were only given the solvable test puzzle and were not given pre-treatment. This method was only applied in the LA noise study; others used the more common line diagram approach.

The line puzzle tasks were conducted in a fixed order of an unsolvable puzzle followed by a solvable puzzle to minimize the negative effects of the task itself. Participants were instructed to trace over lines that interconnect various diagrams of animals (lions, etc.); the objective was to connect all of the diagrams without lifting the pencil or going over any line twice. The measured indicators were the number of attempts to solve the unsolvable puzzle and the time they took persisting with it. The children were instructed to do both puzzles in 10 min and told they should try to solve it until they believed they had solved it or gave up (Munich, Heathrow) or were instructed to keep trying as long as they wanted (ALPINE). The performance on the second solvable task was not taken into account.

The difference between these two approaches was primarily the assumption of when learned helplessness manipulation took place. Within the LA project, it was assumed that children from noisy schools would be more susceptible to helplessness manipulation (by receiving an unsolvable pre-treatment task), would be less likely to solve the test puzzle, slower to find the solution, and more likely to give up. Overall, regardless of treatment task, they were expected to give up more often than quiet-school children.

In the line diagram task, the chronic noise exposure of the children was considered the “pre-treatment.” Here, environmental noise was seen as an unpredictable and uncontrollable factor that could lead to decreased frustration tolerance [24]. The children from noise-exposed schools were expected to have fewer attempts at trying to solve the unsolvable puzzle. The performance on the solvable task was not taken into account.

The results for the learned helplessness tasks are given below.

- *Geometric puzzle task*

The geometric puzzle task was only performed in the LA study. A third of the children assigned to the success pre-treatment task failed to solve the puzzle and, therefore, self-selected into the failure pre-treatment task. Children from noise-exposed schools were more likely to self-select in the failure pre-treatment (*p* < 0.07) [21]. This makes the interpretation of the success-failure pre-treatment effects impossible. However, the comparison between the effect of chronic noise exposure (quiet versus noise-exposed schools) was still made without controlling for their pre-treatment.

Irrespective of the pre-treatment, noise-exposed school children were also more likely to fail the test puzzle (53% of the noise-exposed group failed, 36% of the quiet group, *p* < 0.09) [21]. They were also more likely to give up *p* < 0.001 [21]. Looking within the sample of children who failed to solve the test puzzle, children from noise-exposed schools gave up before their allotted time was up more often (31%) than quiet school children (7%) *p* < 0.025 [21]. The noise-exposed children were less likely to persist at the task than their quiet controls.

The time it took for noise-exposed school children to perform the task was also higher than for quiet school children. The difference in time increased when considering the years enrolled in the school. The time to come to a solution increased from ca.140 s to ca.170 s when the years enrolled went from 2 to 4 years for the exposed group and decreased for the control group from ca.140 to ca.130 when the years enrolled went from 2 to 4 years—however, the interaction time × noise × months was not significant [21].

After wave 1, some of the noise schools received noise abatement measures. At wave 2, the children received no pre-treatment, only a test puzzle. Looking at the cross-sectional results at wave 2, the noise group was more likely to fail (57% failed) than either the abated group (46% failed) or the quiet group (35% failed) *p* < 0.02 [20]. However, for giving up, no differences between the noise and the abated schools were present (noise: 17% gave up, abated: 16% gave up, quiet: 3% gave up) [20]. The longitudinal data provide little evidence that children from the abated schools improved performance. The authors suggest this could be that the effects of previous noise exposure last longer than 1 year or because of other exposures outside the classroom [20].

- *Line diagram task*

The measurement in the line diagram task was the number of attempts taken to try to solve the unsolvable puzzle (persistence). The number of attempts is summarized for all studies in Table 8.

The Heathrow study did not find significant differences in the number of attempts between the exposed and the control group. The ALPINE study only found them when gender was taken into account. Noise had no effect on the motivation of boys, but it did for girls (*p* < 0.05) [26]. The Munich study did find significant differences between children from exposed areas and their controls. Children in the old airport location persisted less than their controls during all three waves, even after the relocation of the airport to the new location [24].

Children in the new airport location started showing significant differences in performance in the third wave, 18 months after the relocation of the airport. They made significantly fewer attempts than their controls (*p* < 0.05) [24].

In the Munich study, the attribution for failure was also measured. Participants had to rate if they attributed their failure at the task to effort, luck, ability, or difficulty. Effort and ability were internal factors, and luck and difficulty were external. In wave 3, the control group at the new location was more likely to attribute failure to difficulty than the exposed group, and the exposed group was more likely to attribute it to ability and luck compared to their quiet counterparts (*p* < 0.05) [24]. So, there was a shift in attribution from difficulty to ability in wave 3 for the newly exposed group. There were no shifts in attribution for failure at the old airport location after relocation.

## 4. Discussion

The findings of the included papers are difficult to compare because of the differences in noise measurement, cognitive tests, and approach to learned helplessness. As this was expected, a meta-analysis was not possible and/or feasible. The quality of the selected papers in regard to the risk of bias is mixed. The longitudinal studies had a higher risk of bias than the cross-sectional ones due to the noise exposure indicators used. Regarding sampling, all studies score high on bias because participants were intentionally selected to represent either an exposed or unexposed group.

### 4.1. Noise

In the four included studies in this review, aircraft noise was the dominant sound source of interest. The noise exposure locations under investigation were the school (LA, Munich, Heathrow) and the home (ALPINE). As all studies concerned children in the primary school age category, it could be expected that they lived relatively close to their school, although this was country-dependent. The Heathrow study gives this as the reason they did not include the effect of the noise levels at home since 80–86% of their sample lived in the same noise contour as their school; therefore, they saw the level at school as day-long exposure [2].

The LA project checked for any differences between children who go to an exposed school but live in a quiet home and children who have both their school and home in an exposed area. No evidence was found for a beneficial effect of having low noise exposure at home when the child went to a noise-exposed school. Differences with the control group (both locations not exposed) were still significant [21].

In the Munich study, participants were selected based on their residency in one of the airport (expected) noise zones; however, only the exposure at their school was measured. In the included papers, there is no control for their noise exposure at home, nor does it state any values of, for example, the noise contours the selection was based on.

The ALPINE study only took into account the noise exposure at home and not at school and did not speculate on any possible combined effect of these exposures. The ALPINE study was also the only study that did not focus on aircraft noise. Instead, the source was general community noise, with the main sources in the area being the road- and rail traffic.

The measurement of noise was diverse in all the included studies, which makes a direct comparison impossible.

### 4.2. Cognitive Tasks and Noise

Although the effects depend on the cognitive test, they show indications that with increasing years of noise exposure, cognitive resources are strained. The LA study reported significant differences in performance in regard to years of enrollment at the school for their visual search task. This can be due to coping mechanisms that are successful in the first years of attending the school but lose their effectiveness over time.

Indications of noise-exposed children adapting this tuning-out coping mechanism are, however, ambiguous. In the Munich study, this theory of a coping mechanism, such as tuning out, does not hold. The Munich study conducted two tasks with an auditory distractor; in both instances, the noise during the task did not contribute to the differences between groups. If the coping mechanism of tuning out had been present, one would expect performance differences between the groups depending on the noise condition during the task and that the exposed group would perform better because they had adapted a tuning out mechanism for environmental noise.

It is noteworthy that the LA study used a human distractor (speech) during the task and the Munich study used broadband noise. Speech sounds are more likely to impair performance than broadband noise. Even when broadband noise is intermittent, speech has meaning and, therefore, is a higher distractor [16]. This may be why effects were found in this study but not in others.

The findings for short-term memory included in this review were not consistent but indicate that short-term memory deficits take some time to develop but also can be restored after the removal of the noise source. The long-term memory and reading tasks showed clearer differences between exposed and non-exposed groups. The Munich study also showed that performance could be recovered after the removal of the noise source. Solely based on the included studies, general conclusions on the relation between noise and cognition cannot be made.

### 4.3. Helplessness and Noise

Looking at similarities between cognition and learned helplessness effects, the performance on both tasks is compared. In the Munich study, both the performance on the cognitive task and the performance on the motivation task (task persistence) decreased over time when environmental noise was introduced. The decreased performance on long-term memory and reading of the newly exposed group gained significance in wave 3. Simultaneously, the lowered task persistence also gained significance in wave 3.

The persistence did not increase for the previously exposed group and remained significantly lower throughout all time points, indicating that the effects of noise on helplessness may be longer lasting.

However, not all studies found helplessness effects. The ALPINE study only found differences in motivation for girls, and because the results for the cognitive tests were not presented differentiating gender, these are difficult to relate. It is known from general learned helplessness literature that women are more vulnerable to helplessness than men [13,30]. The number of attempts of the male group goes against expectation here, with more attempts in the higher exposed group. Overall, the relation between noise exposure and motivation seems less strong in this study than the relation between exposure and memory.

To go deeper into the relationship between cognition and helplessness, the order in which the tests were conducted is relevant as well because helplessness may be moderated by cognitive fatigue. In the Munich and the ALPINE studies, all participants conducted the tasks in the same order, and the motivation task was conducted last (Munich) or second to last (ALPINE). In the LA study, it was unclear when each test was conducted and if the order was fixed for all participants. In the Heathrow study, the tasks were not conducted in a fixed order, as Haines et al. state that group-administered testing was counterbalanced for order and time of day [2].

The reason why the Heathrow study did not find an effect on the motivation task could be that the order of the tasks was counterbalanced. Therefore, not all participants would have the same cognitive fatigue when starting the motivation task. The authors of the Heathrow study also speculated that the puzzle test might measure cognitive effort as a dimension of task persistence [2]. The Munich and the ALPINE studies both conducted the puzzle test at the end of the series of tasks, and they did find a significant difference between the groups. For the LA study, it is not possible to judge the effect of task order since this is not known.

In none of the studies was an analysis done of the interaction between cognitive performance and task persistence. Therefore, it is not known if participants who performed worse at a certain task also performed worse in the motivation task.

In regard to the development of learned helplessness due to environmental noise, the longitudinal studies can inform us. From these two studies, we can see that motivational deficits increased when the duration of exposure to aircraft noise increased and that the problems do not or only marginally lessen when the noise is reduced or removed. The children in the Munich study developed motivation deficits in the new airport location but did recover in the old airport location. In the LA study, the group that received noise abatement measures in their classroom did improve their performance slightly, but the differences with their control group were still significant.

In addition, the Munich study asked participants about their attribution of failure at the task. Exposed children are more likely to attribute failure to stable internal factors like ability or to unstable external conditions such as luck. The children in the new airport location also switched to this attribution in wave 3. From learned helplessness literature, it is known that people more vulnerable to learned helplessness are more likely to have internal and unstable attributions for failure [11,13]. These attributions are, therefore, in line with expectations.

Finally, only the ALPINE study included another variable (gender) in addition to noise to explain the difference in performance on the learned helplessness task. The vulnerability to learned helplessness can also originate from other stressors, physical or social. Therefore, the research could benefit from doing a broader assessment of other known stressors to research a possible cumulative effect.

## 5. Conclusions

In October 2022, a systematic review was completed studying the current evidence of the effect of environmental noise on cognition and helplessness and the interaction between these effects. From the 2443 papers found, 8 were included in the literature review. The main research questions were *how does the sound environment influence the cognitive performance and vulnerability to learned helplessness in childhood to young adulthood, and how are cognitive and helplessness effects related*? The papers included in this systematic review measured the effect of environmental noise on cognition, as well as learned helplessness. They represented four individual studies and concerned mostly with aircraft noise as the environmental noise source. The noise indicators, cognitive tests, and motivational tests varied among the studies making a comparison of the results a challenge. Ultimately, it can be said that there are indications that environmental noise can cause learned helplessness effects, at least in the component of motivation. In this review, these findings are clearer for aircraft noise than other sources because the majority of the papers concerned aircraft noise. Unfortunately, only a small age group was represented, and the effect of noise on different developmental stages of a child cannot be assessed. In the longitudinal studies, the effect on motivation persisted over time, even after the removal of the noise source. This indicates that effects can be lasting, although the time between the measurements is short compared to a child’s life.

The second part of the research question concerned the relationship between cognitive and helplessness effects. For the review, the connection between cognitive and motivational effects is not clear. The performance of reading and long-term memory did decrease in the same time period as motivation did in one of the four studies. This can be an indication of the relationship between motivation and reading and memory tasks, although this is evidence based on only one study. There is some evidence that motivation may be affected by cognitive fatigue as the studies that conducted a fixed order, with the motivation task last, found effects, and the one that conducted them in a random order did not. More consistent research is needed using the same tasks and conditions to assess the development of helplessness and the influence of cognitive performance on it. More in-depth research into differences in age, gender, and social background could also be beneficial to assess the vulnerability to learned helplessness in conjunction with stress factors in the social environment.

## Figures and Tables

**Table 1:** Guidelines for scoring risk for bias [18,19].

Information bias/bias due to exposure assessment	low	Noise level is expressed in *L_den_, L_night_*, or components, AND (a) is based on modelled equivalent noise levels from noise models that used the actual traffic volume, composition, and speed per 24 h per road/railway/airport as input; OR (b) is based on measurements at the façade of the participant’s home and/or school for a minimum of 1 week by qualified staff, and adjusted for data under point (a) as well as meteorological conditions when necessary; OR (c) is based on a noise map reported in a separate publication but which fulfils conditions (a) or (b).
	high	Does not fulfill the conditions mentioned above
	unclear	Not enough information is available to judge the above
Bias due to confounding	low	All important confounders are taken into account, either through matching, restriction, or in the analysis.
	high	Only 1 or no confounder is taken into account OR subjects in exposed and unexposed groups differ for one or more important confounders, and there is no adjustment in the analysis
	unclear	More than one but not all important confounders taken into account OR insufficient information to decide on one of the above.
Bias due to selection of participants	low	Participants randomly sampled from a known population, AND response rate higher than 60%, AND attrition rate less than 20% in follow-up studies.
	high	No random sampling OR response rate less than 60% OR attrition rate higher than 20%
	unclear	No information to judge the above.
Bias due to health outcome assessment	low	The health outcome of interest is objectively measured OR taken from medical records OR taken from questionnaire or interview using a known scale or validated assessment method.
	high	The health outcome of interest is self-reported and not assessed using a known scale or validated assessment method
	unclear	Not sufficient information reported to assess the above.
Bias due to not blinded outcome assessment	low	The health outcome of interest is assessed blind for exposure information in cohort and cross-sectional studies, or exposure is assessed blindly for being a case in case-control studies
	high	The health outcome and/or exposure assessment is not blinded.
	unclear	Not sufficient information reported to assess the above.
Total risk of bias	low	At least 4 at low risk of bias. One “high” or “unclear” out of five is allowed.
	high	Two or more “high” or “unclear”

**Table 2:** Summary of selected papers grouped by project.

Project	References	Study Design	Sample Characteristics	Noise Source	Noise Levels	Cognitive Tests	Learned Helplessness Test
Los Angeles noise project	[20,21]	Longitudinal Tested individually in noise insulated trailer at school Time between measurement waves: 1 year	Wave 1: 262 children Wave 2: 163 children 8–10 years old, gender distribution not stated	Chronic aircraft noise at school	Quantity: indoor sound levels, empty classroom statistical level *L_33_* (wave 2 only), *L_Aeq_* (wave 2 only), and peak levels based on 1 h measurements in morning and afternoon at school. Noise levels at home using CNEL contour levels. Quiet homes < 68 dB CNEL contour Wave 1: Mean peaks Exposed schools 74 dB Mean peaks Quiet schools: 56 dB Wave 2: 43% of the noisy schools received acoustical treatment Mean peaks Exposed schools: 79.1 dB Mean peaks Exposed schools-acoustically treated: 63.2 dB Mean peaks Quiet schools: 56.6 dB	Attention	Puzzle with pieces (triangle, circle, square)
Munich airport study	[22]	Cross-sectional (later extended to longitudinal, in which this paper represents measurement wave 1) Tested individually in noise insulated trailer at school	135 children 8–10 years, gender distribution not stated (no differences exposed and control group)	Chronic aircraft noise living areaAcute during task: induced aircraft or road traffic noise, intermittent broadband noise	Quantity: *L_Aeq24h_* measured outside mobile laboratory at school Means Exposed schools: 68.1 dB(A) Control schools: 59.2 dB(A) Noise during tasks presented at 42–90 dB(A) over headphones	Attention reaction time long-term and short-term Memory Reading	Line diagram puzzles
Munich airport study	[23,24]	Longitudinal Tested individually in noise insulated trailer at schoolMeasurement waves:Wave 1: 6 months before closure of airport Wave 2: 6 months after closure Wave 3: 18 months after closure	326 children 9–13 years, gender distribution not stated (no differences exposed and control group) Fluent German, 2 years residency	Chronic aircraft noise living area Acute during task: induced aircraft or road traffic noise, intermittent broadband noise	Quantity: L_Aeq24h_ measured outside mobile laboratory at school Relocation of the airport at W2. Means in dB(A): Wave 1: Old-noise: 68 dB(A) Old-control: 59 dB(A) New-noise: 53 dB(A) New-control: 53 dB(A) Wave 2/3: Old-noise: 54 dB(A) Old-control: 55 dB(A) New-noise: 62 dB(A) New-control: 55 dB(A)	Attention reaction time long-term and short-term Memory Reading	Line diagram puzzles + attribution of failure
Heathrow study	[2]	Cross-sectional Tested in groups in classroom	340 children 8–10 years, 50% males	Chronic aircraft noise exposure at school acute levels during task: measured indoor levels of aircraft noise	Quantity: 16 h outdoor *L_eq_* from contour maps High-exposed school: Leq_16h_ > 66 dB(A) or Low-exposed schools: Leq_16h_ < 57 dB(A) Acute levels measured during testing	Reading long-term and short-term Memory	Line diagram puzzles
ALPINE	[25,26]	Cross-sectional Tested individually in noise insulated trailer at school	123 children 9–10 years old, 54% male in quiet group, 60% in exposed group	Chronic environmental noise at home–rail and road traffic	Quantity: *L_dn_* and *L_01_*, combination of measured and modeled Mean levels: Quiet homes (<50 dbA): *L_dn_* 46.1 dB(A), *L_01_* 57 dB(A) Exposed homes (>60 dBA): *L_dn_* 62 dB(A), *L_01_* 74 dB(A)	Attention Intentional memory Incidental memory	Line diagram puzzles + Attribution of failure (latter not reported)

**Table 3:** Risk of bias rating for the four studies.

	LA [20,21]	Munich [22,23,24]	Alpine [25,26]	Heathrow [2]
**Information bias/bias due to exposure assessment**	High	High	Low	Low
**Bias due to confounding**	Low	Low	Low	Low
**Bias due to selection of participants**	High	High	High	High
**Bias due to health outcome assessment**	Low	Low	Low	Low
**Bias due to not blinded outcome assessment**	Unclear	Unclear	Low	Low
**Total risk of bias**	High	High	Low	Low

**Table 4:** Task performance results of the exposed group compared to the non-exposed control group. Arrows indicating lower or higher performance, size indicating full (large arrow) or partial (small arrow) relationship, colors indicating if the differences are positive (green) or negative (red) for the exposed group. Yellow stripe indicates no differences between groups.

		Cognition	Helplessness Measured in Motivation
Longitudinal	LA noise project	Attention: Visual search task <2 years of exposure 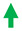 >4 years of exposure 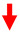	Ability to solve puzzle 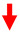 Persistence at task 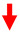 Time to complete task 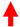
	Munich airport	Attention: Visual search task 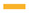 Attention: Reaction time 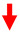 Memory: long-term 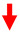 Memory: short-term 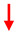 Only for a part of the test and not all waves Reading 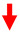	Persistence in no attempts 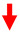 Attribution of failure: internal
Cross-sectional	Alpine	Attention: Visual search task 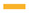 Memory: intentional 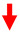 Memory: incidental 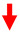	Persistence in no attempts 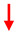 *Girls only*
	Heathrow airport	Reading 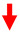 Memory: long-term 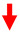 Memory: short-term 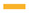	Persistence in no attempts 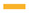

**Table 5:** Visual search tasks per study and their outcomes.

	Test	Noise Condition during Task	Noise Exposure Groups	Performance on Task (Mean Score/Max. Score)
LA [20,21]	Crossing out Es in a text with and without a distraction. Timed at 2 min. percentage of E’s found in distraction task adjusted for no distraction performance	Without distraction: quiet noise-insulated trailer With distraction: male voice reading a story on moderate volume over headphones, no environmental noise	Measurement wave 1: Mean peak low noise 56 dB(A)	<2 years enrolled: 85.4/100 >4 years enrolled: 89.2/100
			Measurement Wave 1: Mean peak high noise 74 dB(A)	<2 years enrolled: 88.1/100 >4 years enrolled: 85.9/100
			Measurement wave 2: Mean peak low noise 56 dB(A)	<2 years enrolled: 89.6/100 >4 years enrolled: 90.3/100
			Measurement wave 2: Mean peak high noise 79 dB(A)	<2 years enrolled: 90.6/100 >4 years enrolled: 90/100
Munich [22,23]	Searching for 5 simple target figures within 12 complex figures. Measure number of correct identifications	Quiet, sound-attenuated trailer	Old airport location-quiet control *L_Aeq_* 59 dB(A)	5.6/12 *
			Old airport location-noise-exposed *L_Aeq_* 68 dB(A)	6.1/12 *
ALPINE [25]	Line drawings of fish, task is to circle fish facing in the opposite direction of the others. Timed at 2 min. measure is number of correctly marked fish out of 23	Quiet, sound-attenuated trailer	Neighborhoods *L_dn_* <50 dB(A)	21.6/23
			Neighborhoods *L_dn_* > 60 dB(A)	21.55/23

* Only known for measurement wave 1.

**Table 6:** Reaction time tasks per study and their outcomes.

	Test	Noise Condition during Task	Noise Exposure Groups	Performance on Task *
Munich [22]	Respond to occurrences of colored light by pressing a button of the corresponding color. With and without distraction for 2 × 5 min (wave 1) or 2 × 8 min (wave 2, 3)	Quiet, sound-attenuated trailer And Aircraft noise at 80 dB(A) (wave 1) or 85 dB(A) (wave 2, 3)	Measurement Wave 1 Old airport location-quiet control L_Aeq_ 59 dB(A) Old airport location-noise-exposed L_Aeq_ 68 dB(A)	Old-quiet 440.7 ms Old-noise 450.0 ms Noisy conditions Old-quiet 438.0 ms Old-noise 454.0 ms

* Only known for wave 1.

**Table 7:** Memory tasks per study.

	Test	Noise Condition during Task	Noise Exposure Groups
Munich [22,23]	Short term: Consonants presented at 1/s rate over headphones, stopped randomly. Recall as many consonants in order starting from the end of the sequence. Measure number of letters in correct or adjacent position. Long term: Reading a text under noise conditions for 12 min. Recall tested after 1 day using six multiple choice questions and three recall questions	Short-term: Quiet, sound-attenuated trailer Long-term: intermittent broadband noise bursts peak 80 dBA during the retention period	Measurement wave 1 Old-noise *L_Aeq_* 68 dB(A) Old-quiet *L_Aeq_* 59 dB(A) New-noise *L_Aeq_* 62 dB(A) New-quiet *L_Aeq_* 55 dB(A) Measurement wave 2 Old-noise *L_Aeq_* 54 dB(A) Old-quiet *L_Aeq_* 55 dB(A) New-noise *L_Aeq_* 62- dB(A) New-quiet *L_Aeq_* 55 dB(A)
ALPINE [25]	Intentional memory: the ability to recall and complete sentences from a story after 10 min. Incidental memory: free recall (name as many as you can remember) and recognition of the line diagrams used in the motivational test amongst similar line diagrams.	Short-term: Quiet, sound-attenuated trailer	Neighborhoods *L_dn_* < 50 dB(A) Neighborhoods *L_dn_* > 60 dB(A)
Heathrow [2]	Short term: Six trials of serial digit recall (5, 7, and 9 digits) sum of the averages scores per length Reading a text under quiet conditions, time not specified. Recall tested after 1 day using six multiple choice questions and three recall questions.	Short-term: Monitored environment, inside classroom Long-term: Monitored environment, inside classroom	High noise 66 dB(A)contour *L_Aeq_* Low noise outside 57 dB(A)contour

**Table 8:** Results for task persistence of line diagram tasks per study.

		Noise Exposure Groups	Mean No. Attempts	
Munich [22,23,24]	Measurement wave 1	Old-noise *L_Aeq_* 68 dB(A)	5.4	
		Old-quiet *L_Aeq_* 59 dB(A)	6.5	
		New-noise *L_Aeq_* 62 dB(A)	5.7	
		New-quiet *L_Aeq_* 55 dB(A)	5.8	
	Measurement wave 2	Old-noise *L_Aeq_* 54 dB(A)	7.4	
		Old-quiet *L_Aeq_* 55 dB(A)	8.8	
		New-noise *L_Aeq_* 62 dB(A)	7.3	
		New-quiet *L_Aeq_* 55 dB(A)	7.2	
	Measurement wave 3	Old-noise *L_Aeq_* 54 dB(A)	6.8	
		Old-quiet *L_Aeq_* 55 dB(A)	7.9	
		New-noise *L_Aeq_* 62 dB(A)	6.3	
		New-quiet *L_Aeq_* 55 dB(A)	7.9	
ALPINE [25,26]		Neighborhoods *L_dn_* < 50 dB(A)	Girls	5.5
			Boys	5.54
		Neighborhoods *L_dn_* > 60 dB(A)	Girls	4.26
			Boys	4.91
Heathrow [2]		High noise > 66 dB(A)contour	5.86	
		Low noise < 57 dB(A)contour	5.93	

## Data Availability

Not applicable.

## References

[1] Evans G.W. (2006). Child Development and the Physical Environment. Annu. Rev. Psychol..

[2] Haines M.M., Stansfeld S.A., Job R.F.S., Berglund B., Head J. (2001). Chronic aircraft noise exposure, stress responses, mental health and cognitive performance in school children. Psychol. Med..

[3] Hughes R.W. (2014). Auditory distraction: A duplex-mechanism account. PsyCh J..

[4] Cohen S., Evans G.W., Stokols D., Krantz D.S. (1986). Behavior, Health, and Environmental Stress.

[5] Massonnié J., Mareschal D., Kirkham N.Z. (2022). Individual differences in dealing with classroom noise disturbances. Mind Brain Educ..

[6] Vasilev M.R., Kirkby J.A., Angele B. (2018). Auditory Distraction During Reading: A Bayesian Meta-Analysis of a Continuing Controversy. Perspect. Psychol. Sci..

[7] Leist L., Lachmann T., Klatte M. Differential Effects of Irrelevant Speech and Environmental Sounds on Short-Term Memory in Children and Adults. Proceedings of the ICBEN Conference.

[8] Lazarus R.S. (1966). Psychological Stress and the Coping Process.

[9] Lazarus R.S., Folkman S. (1984). Stress, Appraisal, and Coping.

[10] Maier S.F., Seligman M.E. (1976). Learned helplessness: Theory and evidence. J. Exp. Psychol. Gen..

[11] Abramson L.Y., Seligman M.E., Teasdale J.D. (1978). Learned helplessness in humans: Critique and reformulation. J. Abnorm. Psychol..

[12] Evans G.W., Stecker R. (2004). Motivational consequences of environmental stress. J. Environ. Psychol..

[13] Glass D.C., Singer J.E. (1972). Urban Stress: Experiments on Noise and Social Stressors.

[14] Basner M., Babisch W., Davis A., Brink M., Clark C., Janssen S., Stansfeld S. (2014). Auditory and non-auditory effects of noise on health. Lancet.

[15] Clark C., Paunovic K. (2018). WHO Environmental Noise Guidelines for the European Region: A Systematic Review on Environmental Noise and Quality of Life, Wellbeing and Mental Health. Int. J. Environ. Res. Public Health.

[16] Klatte M., Bergström K., Lachmann T. (2013). Does noise affect learning? A short review on noise effects on cognitive performance in children. Front. Psychol..

[17] Stansfeld S., Clark C. (2015). Health Effects of Noise Exposure in Children. Curr. Environ. Health Rep..

[18] van Kempen E., Casas M., Pershagen G., Foraster M. (2017). Cardiovascular and metabolic effects of environmental noise Systematic evidence review in the framework of the development of the WHO environmental noise guidelines for the European Region. RIVM Report 2017-0078.

[19] World Health Organization (2012). WHO Handbook for Guideline Development.

[20] Cohen S., Krantz D.S., Evans G.W., Stokols D., Kelly S. (1981). Aircraft noise and children: Longitudinal and cross-sectional evidence on adaptation to noise and the effectiveness of noise abatement. J. Pers. Soc. Psychol..

[21] Cohen S., Evans G.W., Krantz D.S., Stokols D. (1980). Physiological, motivational, and cognitive effects of aircraft noise on children: Moving from the laboratory to the field. Am. Psychol..

[22] Evans G.W., Hygge S., Bullinger M. (1995). Chronic Noise and Psychological Stress. Psychol. Sci..

[23] Hygge S., Evans G.W., Bullinger M. (2002). A Prospective Study of Some Effects of Aircraft Noise on Cognitive Performance in Schoolchildren. Psychol. Sci..

[24] Bullinger M., Hygge S., Evans G.W., Meis M., Mackensen S.V. (1999). The Psychological Cost of Aircraft Noise for Children. Zent. Hyg. Umweltmed..

[25] Lercher P., Evans G.W., Meis M. (2003). Ambient Noise and Cognitive Processes among Primary Schoolchildren. Environ. Behav..

[26] Evans G.W., Lercher P., Meis M., Ising H., Kofler W.W. (2001). Community noise exposure and stress in children. J. Acoust. Soc. Am..

[27] World Health Organization (2014). Handbook for Guideline Development.

[28] Civil Aviation Authority Measuring and Modelling Noise. https://www.caa.co.uk/consumers/environment/noise/measuring-and-modelling-noise/.

[29] Biglmaier F. (1969). Die Lesetest Serie.

[30] Dweck C., Elliot E. (1983). Achievement Motivation. Handbook of Child Psychology and Developmental Science.

